# 2,6-Dihy­droxy-4-oxo-2-(pyridin-1-ium-3-yl)-4*H*-1,3,2-benzodioxaborinin-2-ide 0.67-hydrate

**DOI:** 10.1107/S1600536814004267

**Published:** 2014-03-05

**Authors:** Blanca A. Garcia-Grajeda, Herbert Höpfl, Jorge A. Guerrero-Alvarez, José J. Campos-Gaxiola, Adriana Cruz-Enríquez

**Affiliations:** a Facultad de Ingenieria Mochis, Universidad Autónoma de Sinaloa, Fuente Poseidón y Prol. A. Flores S/N, CP 81223, C.U. Los Mochis, Sinaloa, México; b Centro de Graduados e Investigación en Química, Universidad Autónoma del Estado de Morelos, Av. Universidad 1001, CP 62210, Cuernavaca, Morelos, México

## Abstract

The asymmetric unit of the title compound, C_12_H_10_BNO_5_·0.67H_2_O, contains three independent pyridinylboronic acid esters adopting zwitterionic forms and two water mol­ecules. The six-membered heterocyclic rings in the boronic esters have half-chair conformations and the deviations of the B atoms from the boronate mean planes range from 0.456 (3) to 0.657 (3) Å. All of the B atoms have tetra­hedral coordination environments, with B—O and B—C bond lengths of 1.446 (4)–1.539 (3) and 1.590 (5)–1.609 (5) Å, respectively. In the crystal, the ester and water mol­ecules are linked into a three-dimensional network by a large number of O—H⋯O, N—H⋯O and C—H⋯O hydrogen bonds. The crystal packing is further accomplished by π–π inter­actions, with centroid–centroid distances of 3.621 (4)–3.787 (4) Å.

## Related literature   

For the synthesis and applications of boronic esters, see: Höpfl (2002 ▶); Fujita *et al.* (2008 ▶); Severin (2009 ▶). For related structures, see: Barba *et al.* (2010 ▶).
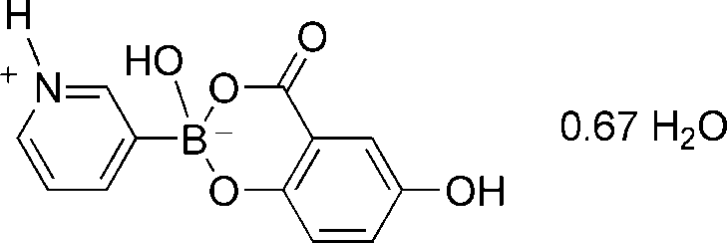


## Experimental   

### 

#### Crystal data  

C_12_H_10_BNO_5_·0.67H_2_O
*M* *_r_* = 271.03
Triclinic, 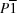

*a* = 10.350 (2) Å
*b* = 13.916 (3) Å
*c* = 14.340 (3) Å
α = 65.785 (4)°
β = 73.421 (4)°
γ = 87.213 (5)°
*V* = 1799.8 (7) Å^3^
*Z* = 6
Mo *K*α radiation
μ = 0.12 mm^−1^
*T* = 100 K
0.45 × 0.41 × 0.28 mm

#### Data collection  

Bruker APEX CCD area-detector diffractometer
Absorption correction: multi-scan (*SADABS*; Sheldrick, 1996 ▶) *T*_min_ = 0.95, *T*_max_ = 0.97
18938 measured reflections
7051 independent reflections
3529 reflections with *I* > 2σ(*I*)
*R*_int_ = 0.092

#### Refinement  

*R*[*F* ^2^ > 2σ(*F* ^2^)] = 0.053
*wR*(*F* ^2^) = 0.092
*S* = 1.02
7051 reflections
571 parameters
13 restraints
H atoms treated by a mixture of independent and constrained refinement
Δρ_max_ = 0.71 e Å^−3^
Δρ_min_ = −0.26 e Å^−3^

### 

Data collection: *SMART* (Bruker, 2000 ▶); cell refinement: *SAINT-Plus-NT* (Bruker, 2001 ▶); data reduction: *SAINT-Plus-NT*; program(s) used to solve structure: *SHELXTL-NT* (Sheldrick, 2008 ▶); program(s) used to refine structure: *SHELXTL-NT*; molecular graphics: *ORTEP-3 for Windows* (Farrugia, 2012 ▶) and *Mercury* (Macrae *et al.*, 2008 ▶); software used to prepare material for publication: *publCIF* (Westrip, 2010 ▶).

## Supplementary Material

Crystal structure: contains datablock(s) I, New_Global_Publ_Block. DOI: 10.1107/S1600536814004267/is5341sup1.cif

Structure factors: contains datablock(s) I. DOI: 10.1107/S1600536814004267/is5341Isup2.hkl

CCDC reference: 988588

Additional supporting information:  crystallographic information; 3D view; checkCIF report

## Figures and Tables

**Table 1:** Hydrogen-bond geometry (Å, °)

*D*—H⋯*A*	*D*—H	H⋯*A*	*D*⋯*A*	*D*—H⋯*A*
O62—H62*A*⋯O25	0.84	1.86	2.702 (4)	174
O45—H45′⋯O23^i^	0.84	1.94	2.777 (2)	177
O4—H4′⋯O45^ii^	0.84	1.77	2.579 (3)	162
O61—H61*A*⋯O43^ii^	0.84	1.95	2.749 (3)	159
O5—H5′⋯O3^iii^	0.84	1.93	2.773 (2)	178
O24—H24′⋯O5^iv^	0.84	1.8	2.638 (3)	179
O25—H25′⋯O4^v^	0.84	1.95	2.791 (3)	173
O62—H62*B*⋯O24^vi^	0.84	2.02	2.810 (4)	157
O44—H44′⋯O61^vii^	0.84	1.82	2.656 (3)	177
O61—H61*B*⋯O62^viii^	0.84	1.83	2.673 (3)	178
N1—H1′⋯O23^i^	0.84	1.89	2.725 (4)	176
N21—H21′⋯O3^ix^	0.84	1.89	2.727 (4)	170
N41—H41′⋯O43^x^	0.84	1.92	2.749 (5)	169
C11—H11⋯O62^iv^	0.95	2.57	3.348 (4)	140
C23—H23⋯O5^iv^	0.95	2.59	3.215 (4)	123
C43—H43⋯O61^vii^	0.95	2.57	3.201 (4)	124

Symmetry codes: (i) 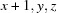; (ii) 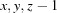; (iii) 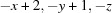; (iv) 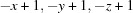; (v) 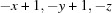; (vi) 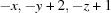; (vii) 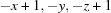; (viii) 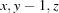; (ix) 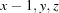; (x) 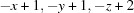.

**Fig. 1.**
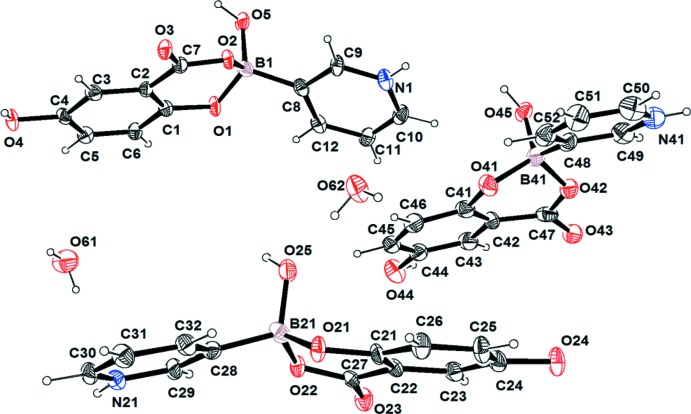
Molecular structures in the asymmetric unit of the title compound, with the atom-labelling. Displacement ellipsoids are drawn at the 50% probability level.

**Fig. 2.**
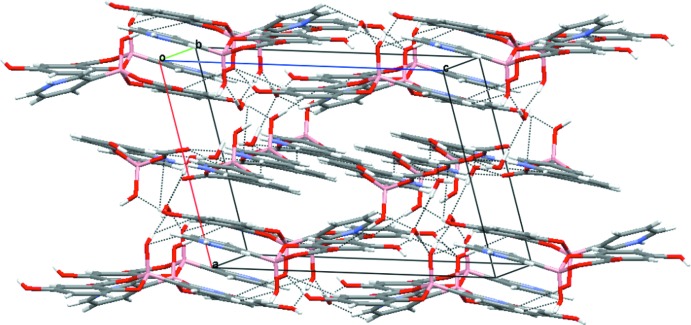
A crystal packing diagram of the title compound viewed along the b axis, showing the three-dimensional hydrogen-bonded network. Hydrogen bonds were drawn as dashed lines.

## supplementary crystallographic information

### 1. Comment

The O—H groups of boronic acids are able to react with alcohols to give boronic esters through the formation of covalent B–O bonds (Fujita *et al.*, 2008). The high thermodynamic stability of boronic esters has been employed for the formation of *cyclo*-oligomeric and polymeric boron compounds with potential applications in gas storage and separation (Höpfl *et al.*, 2002; Severin *et al.*, 2009). Herein, we report on the solid-state structure of a new boronic ester formed between 2,5-dihydroxybenzoic acid and 3-pyridine boronic acid.

The molecular components of the title compound, 6-hydroxy-2-(pyridinium-3-yl)-4*H*-benzo-1,3,2-dioxaborininato-4-one) 0.67-hydrate, are shown in Fig. 1. The asymmetric unit contains three crystallographically independent ester molecules with similar conformations and two water molecules. The six-membered heterocyclic rings in the boronic esters have half-chair conformations, and the deviation of the B atom from the boronate mean planes range from 0.456 (3) to 0.657 (3) Å. The B atoms have tetrahedral coordination environments with B—O and B—C bond distances of 1.446 (4)–1.539 (3) Å and 1.590 (5)–1.609 (5) Å, respectively. The tetrahedral character of the boron atoms was evidenced also by ^11^B-NMR spectroscopy, giving a chemical shift of 3.8 p.p.m. (Barba *et al.*, 2010). In the crystal, the molecular entities are linked into a three-dimensional network by a large number of O—H···O and N^+^—H···^-^O hydrogen bonds. Crystal packing is accomplished by additional C—H···O and *π*–*π* contacts (Table 1 and Fig 2), of which the latter are formed between pyridinium and phenyl rings. The centroid-centroid distances are *Cg*1···*Cg*2^i^ = 3.774 (4) Å, *Cg*1···*Cg*4^ii^ = 3.787 (4) Å and *Cg*2···*Cg*3^iii^ = 3.621 (4) Å, where *Cg*1, *Cg*2, *Cg*3 and *Cg*4 are the centroids of C1–C6, N21/C28–C32, C41–C46 and N41/C48–C52 rings, respectively. [symmetry codes: (i) 1 - *x*, 1 - *y*, -*z*; (ii) 1 - *x*, 1 - *y*, 1 - *z*; (iii) -*x*, 1 - *y*, 1 - *z*.]

### 2. Experimental

C_12_H_10_BNO_5_ was formed from a solution of 3-pyridin boronic acid (0.05 g, 0.41 mmol), 2,5-dihydroxybenzoic acid (0.06 g, 0.41 mmol) in a solvent mixture of CH_3_OH (8 ml) and H_2_O (2 ml), which was heated under reflux for 1 h, giving a clear transparent solution. Cooling the reaction mixture slowly to room temperature afforded yellow crystals suitable for X-ray diffraction in approximately 55% yield. ^11^B NMR (64 MHz, DMSO-d_6_) δ: 3.8 p.p.m..

### 3. Refinement

H atoms bonded to C atoms were positioned geometrically and constrained using the riding-model approximation [C—H = 0.95 A and *U*_iso_(H) = 1.2*U*_eq_(C)]. H atoms bonded to O and N were initially located in a difference Fourier map; then, the positions were refined with O(N)—H distance restraints of 0.840 (1) A and with *U*_iso_(*H*) = 1.5*U*_eq_(O,N).

### Crystal data

**Table:**

C_12_H_10_BNO_5_·0.67H_2_O	*Z* = 6
*M**_r_* = 271.03	*F*(000) = 844
Triclinic, *P*1	*D*_x_ = 1.500 Mg m^−^^3^
Hall symbol: -P 1	Mo *K*α radiation, λ = 0.71073 Å
*a* = 10.350 (2) Å	Cell parameters from 1641 reflections
*b* = 13.916 (3) Å	θ = 2.5–21.2°
*c* = 14.340 (3) Å	µ = 0.12 mm^−^^1^
α = 65.785 (4)°	*T* = 100 K
β = 73.421 (4)°	Block, light yellow
γ = 87.213 (5)°	0.45 × 0.41 × 0.28 mm
*V* = 1799.8 (7) Å^3^	

### Data collection

**Table:**

Bruker APEX CCD area-detector diffractometer	7051 independent reflections
Radiation source: fine-focus sealed tube	3529 reflections with *I* > 2σ(*I*)
Graphite monochromator	*R*_int_ = 0.092
Detector resolution: 8.3 pixels mm^-1^	θ_max_ = 26.0°, θ_min_ = 1.8°
phi and ω scans	*h* = −12→12
Absorption correction: multi-scan (*SADABS*; Sheldrick, 1996)	*k* = −17→17
*T*_min_ = 0.95, *T*_max_ = 0.97	*l* = −17→17
18938 measured reflections	

### Refinement

**Table:**

Refinement on *F*^2^	Primary atom site location: structure-invariant direct methods
Least-squares matrix: full	Secondary atom site location: difference Fourier map
*R*[*F*^2^ > 2σ(*F*^2^)] = 0.053	Hydrogen site location: inferred from neighbouring sites
*wR*(*F*^2^) = 0.092	H atoms treated by a mixture of independent and constrained refinement
*S* = 1.02	*w* = 1/[σ^2^(*F*_o_^2^) + (0.0125*P*)^2^] where *P* = (*F*_o_^2^ + 2*F*_c_^2^)/3
7051 reflections	(Δ/σ)_max_ < 0.001
571 parameters	Δρ_max_ = 0.71 e Å^−^^3^
13 restraints	Δρ_min_ = −0.26 e Å^−^^3^

### Special details

**Table:**

Experimental. IR (KBr): 3520, 3370, 2950, 3024, 2942, 1648, 1615, 1562, 1480, 1305 and 786 cm^-1^.
Geometry. All e.s.d.'s (except the e.s.d. in the dihedral angle between two l.s. planes) are estimated using the full covariance matrix. The cell e.s.d.'s are taken into account individually in the estimation of e.s.d.'s in distances, angles and torsion angles; correlations between e.s.d.'s in cell parameters are only used when they are defined by crystal symmetry. An approximate (isotropic) treatment of cell e.s.d.'s is used for estimating e.s.d.'s involving l.s. planes.
Refinement. Refinement of *F*^2^ against ALL reflections. The weighted *R*-factor *wR* and goodness of fit *S* are based on *F*^2^, conventional *R*-factors *R* are based on *F*, with *F* set to zero for negative *F*^2^. The threshold expression of *F*^2^ > σ(*F*^2^) is used only for calculating *R*-factors(gt) *etc*. and is not relevant to the choice of reflections for refinement. *R*-factors based on *F*^2^ are statistically about twice as large as those based on *F*, and *R*- factors based on ALL data will be even larger.

### Fractional atomic coordinates and isotropic or equivalent isotropic displacement parameters (Å^2^)

**Table:**

	*x*	*y*	*z*	*U*_iso_*/*U*_eq_	
B1	0.9002 (3)	0.3451 (3)	0.2383 (3)	0.0221 (9)	
N1	0.8273 (3)	0.4137 (2)	0.4822 (2)	0.0249 (7)	
H1'	0.837 (3)	0.4691 (13)	0.490 (2)	0.037*	
O1	0.83252 (17)	0.25852 (15)	0.23193 (14)	0.0223 (5)	
O2	0.86471 (17)	0.45237 (15)	0.16481 (14)	0.0196 (5)	
O3	0.82959 (17)	0.55863 (15)	0.01161 (15)	0.0204 (5)	
O4	0.76584 (19)	0.30256 (15)	−0.15147 (16)	0.0230 (5)	
H4'	0.752 (3)	0.3656 (7)	−0.185 (2)	0.034*	
O5	1.04678 (19)	0.34308 (16)	0.20681 (14)	0.0237 (5)	
H5'	1.083 (2)	0.374 (2)	0.1405 (4)	0.036*	
C1	0.8181 (3)	0.2723 (3)	0.1362 (2)	0.0210 (8)	
C2	0.8197 (2)	0.3732 (2)	0.0568 (2)	0.0162 (7)	
C3	0.8017 (2)	0.3854 (2)	−0.0408 (2)	0.0195 (7)	
H3	0.8042	0.4540	−0.0954	0.023*	
C4	0.7803 (3)	0.2968 (2)	−0.0568 (2)	0.0189 (8)	
C5	0.7774 (2)	0.1964 (2)	0.0234 (2)	0.0195 (7)	
H5	0.7624	0.1359	0.0118	0.023*	
C6	0.7958 (3)	0.1834 (2)	0.1190 (2)	0.0203 (8)	
H6	0.7935	0.1145	0.1730	0.024*	
C7	0.8381 (3)	0.4674 (3)	0.0756 (2)	0.0207 (8)	
C8	0.8476 (3)	0.3359 (2)	0.3585 (2)	0.0204 (8)	
C9	0.8663 (3)	0.4209 (2)	0.3818 (2)	0.0258 (8)	
H9	0.9080	0.4860	0.3250	0.031*	
C10	0.7715 (3)	0.3239 (3)	0.5661 (2)	0.0249 (8)	
H10	0.7465	0.3211	0.6363	0.030*	
C11	0.7504 (3)	0.2356 (3)	0.5504 (2)	0.0252 (8)	
H11	0.7115	0.1709	0.6093	0.030*	
C12	0.7875 (3)	0.2433 (2)	0.4464 (2)	0.0223 (8)	
H12	0.7711	0.1830	0.4349	0.027*	
B21	−0.0548 (3)	0.8085 (3)	0.2594 (3)	0.0228 (9)	
N21	−0.1361 (2)	0.7302 (2)	0.0507 (2)	0.0203 (6)	
H21'	−0.147 (3)	0.6730 (11)	0.046 (2)	0.030*	
O21	−0.12591 (17)	0.88671 (15)	0.29523 (15)	0.0223 (5)	
O22	−0.09568 (17)	0.69622 (15)	0.34851 (15)	0.0217 (5)	
O23	−0.14161 (18)	0.58725 (16)	0.51874 (15)	0.0241 (5)	
O24	−0.14430 (19)	0.83893 (16)	0.70031 (16)	0.0263 (5)	
H24'	−0.113 (3)	0.7810 (11)	0.729 (2)	0.039*	
O25	0.08854 (18)	0.83243 (16)	0.23529 (16)	0.0241 (5)	
H25'	0.139 (2)	0.7942 (19)	0.210 (2)	0.036*	
C21	−0.1338 (3)	0.8719 (3)	0.3963 (2)	0.0206 (8)	
C22	−0.1302 (3)	0.7722 (2)	0.4743 (2)	0.0178 (7)	
C23	−0.1363 (3)	0.7592 (2)	0.5777 (2)	0.0212 (8)	
H23	−0.1352	0.6907	0.6312	0.025*	
C24	−0.1440 (3)	0.8462 (2)	0.6014 (2)	0.0213 (8)	
C25	−0.1537 (3)	0.9453 (2)	0.5241 (2)	0.0255 (8)	
H25	−0.1638	1.0045	0.5419	0.031*	
C26	−0.1489 (3)	0.9593 (2)	0.4221 (2)	0.0245 (8)	
H26	−0.1559	1.0273	0.3701	0.029*	
C27	−0.1224 (3)	0.6792 (3)	0.4487 (2)	0.0199 (8)	
C28	−0.0973 (3)	0.8118 (2)	0.1587 (2)	0.0182 (7)	
C29	−0.1085 (3)	0.7234 (2)	0.1394 (2)	0.0213 (8)	
H29	−0.0965	0.6561	0.1900	0.026*	
C30	−0.1534 (3)	0.8226 (2)	−0.0245 (2)	0.0212 (8)	
H30	−0.1701	0.8247	−0.0871	0.025*	
C31	−0.1470 (3)	0.9131 (2)	−0.0107 (2)	0.0251 (8)	
H31	−0.1602	0.9790	−0.0629	0.030*	
C32	−0.1203 (2)	0.9070 (2)	0.0823 (2)	0.0216 (8)	
H32	−0.1180	0.9696	0.0934	0.026*	
B41	0.5523 (3)	0.5007 (3)	0.7575 (3)	0.0271 (10)	
N41	0.5362 (3)	0.7370 (3)	0.8323 (2)	0.0331 (7)	
H41'	0.549 (3)	0.746 (3)	0.8832 (17)	0.050*	
O41	0.47752 (18)	0.48464 (17)	0.69074 (15)	0.0278 (6)	
O42	0.49835 (18)	0.42130 (17)	0.87233 (15)	0.0253 (5)	
O43	0.44602 (18)	0.25267 (16)	0.98428 (16)	0.0293 (6)	
O44	0.4175 (2)	0.07153 (18)	0.74251 (18)	0.0370 (6)	
H44'	0.452 (3)	0.035 (2)	0.7914 (17)	0.056*	
O45	0.69516 (18)	0.47905 (17)	0.72686 (15)	0.0255 (5)	
H45'	0.743 (2)	0.514 (2)	0.6640 (8)	0.038*	
C41	0.4587 (3)	0.3823 (3)	0.7069 (2)	0.0257 (8)	
C42	0.4645 (3)	0.2998 (3)	0.8004 (2)	0.0222 (8)	
C43	0.4520 (3)	0.1950 (3)	0.8145 (2)	0.0275 (8)	
H43	0.4589	0.1389	0.8786	0.033*	
C44	0.4293 (3)	0.1734 (3)	0.7338 (2)	0.0257 (8)	
C45	0.4154 (3)	0.2559 (3)	0.6422 (2)	0.0277 (8)	
H45	0.3957	0.2411	0.5884	0.033*	
C46	0.4301 (3)	0.3593 (3)	0.6284 (2)	0.0277 (8)	
H46	0.4207	0.4153	0.5652	0.033*	
C47	0.4713 (3)	0.3225 (3)	0.8912 (3)	0.0275 (8)	
C48	0.5329 (3)	0.6165 (3)	0.7528 (2)	0.0245 (8)	
C49	0.5463 (3)	0.6392 (3)	0.8358 (3)	0.0300 (9)	
H49	0.5629	0.5839	0.8965	0.036*	
C50	0.5167 (3)	0.8196 (3)	0.7474 (3)	0.0361 (9)	
H50	0.5147	0.8887	0.7456	0.043*	
C51	0.4998 (3)	0.8035 (3)	0.6645 (3)	0.0350 (9)	
H51	0.4834	0.8607	0.6050	0.042*	
C52	0.5070 (3)	0.7020 (3)	0.6678 (2)	0.0309 (9)	
H52	0.4938	0.6907	0.6100	0.037*	
O61	0.4664 (2)	0.0441 (2)	0.10850 (19)	0.0460 (7)	
H61A	0.468 (3)	0.1027 (14)	0.0576 (19)	0.069*	
H61B	0.3878 (13)	0.028 (3)	0.151 (2)	0.069*	
O62	0.2191 (2)	0.98825 (19)	0.24779 (19)	0.0329 (6)	
H62A	0.175 (3)	0.9429 (18)	0.242 (3)	0.049*	
H62B	0.175 (3)	1.0343 (18)	0.264 (2)	0.049*	

### Atomic displacement parameters (Å^2^)

**Table:**

	*U*^11^	*U*^22^	*U*^33^	*U*^12^	*U*^13^	*U*^23^
B1	0.024 (2)	0.019 (2)	0.021 (2)	−0.0021 (17)	−0.0106 (18)	−0.0041 (19)
N1	0.0314 (16)	0.026 (2)	0.0243 (17)	0.0072 (15)	−0.0157 (13)	−0.0130 (16)
O1	0.0317 (12)	0.0254 (14)	0.0138 (13)	0.0026 (10)	−0.0129 (10)	−0.0079 (10)
O2	0.0264 (12)	0.0219 (13)	0.0147 (12)	0.0037 (9)	−0.0125 (10)	−0.0075 (10)
O3	0.0280 (12)	0.0197 (13)	0.0141 (12)	0.0034 (10)	−0.0092 (10)	−0.0058 (11)
O4	0.0318 (12)	0.0257 (14)	0.0190 (14)	0.0090 (11)	−0.0165 (10)	−0.0112 (11)
O5	0.0267 (13)	0.0311 (15)	0.0120 (12)	0.0024 (10)	−0.0094 (10)	−0.0053 (11)
C1	0.0194 (17)	0.028 (2)	0.0165 (19)	−0.0002 (15)	−0.0074 (14)	−0.0081 (17)
C2	0.0154 (16)	0.020 (2)	0.0141 (18)	0.0028 (14)	−0.0050 (14)	−0.0079 (16)
C3	0.0164 (16)	0.022 (2)	0.0201 (19)	0.0022 (14)	−0.0088 (14)	−0.0060 (16)
C4	0.0155 (17)	0.025 (2)	0.0198 (19)	0.0054 (15)	−0.0106 (14)	−0.0099 (17)
C5	0.0207 (17)	0.018 (2)	0.022 (2)	0.0044 (14)	−0.0067 (15)	−0.0103 (16)
C6	0.0231 (17)	0.021 (2)	0.0157 (19)	0.0023 (15)	−0.0076 (14)	−0.0057 (16)
C7	0.0136 (17)	0.032 (2)	0.019 (2)	0.0037 (15)	−0.0046 (14)	−0.0138 (18)
C8	0.0208 (17)	0.024 (2)	0.022 (2)	0.0052 (15)	−0.0130 (15)	−0.0097 (17)
C9	0.0336 (19)	0.033 (2)	0.0109 (19)	0.0014 (16)	−0.0128 (16)	−0.0048 (17)
C10	0.0254 (18)	0.033 (2)	0.0141 (19)	0.0068 (17)	−0.0088 (15)	−0.0067 (18)
C11	0.0285 (18)	0.028 (2)	0.0155 (19)	−0.0004 (16)	−0.0064 (15)	−0.0049 (16)
C12	0.0253 (18)	0.024 (2)	0.025 (2)	0.0060 (15)	−0.0132 (15)	−0.0132 (17)
B21	0.024 (2)	0.022 (2)	0.024 (2)	0.0031 (18)	−0.0151 (18)	−0.0059 (19)
N21	0.0208 (14)	0.0213 (18)	0.0225 (16)	0.0019 (13)	−0.0085 (12)	−0.0112 (15)
O21	0.0301 (12)	0.0268 (14)	0.0156 (13)	0.0106 (10)	−0.0124 (10)	−0.0112 (11)
O22	0.0255 (12)	0.0278 (14)	0.0134 (13)	0.0019 (10)	−0.0085 (10)	−0.0083 (11)
O23	0.0333 (12)	0.0204 (14)	0.0172 (13)	−0.0001 (10)	−0.0096 (10)	−0.0049 (11)
O24	0.0406 (14)	0.0272 (15)	0.0187 (14)	0.0121 (11)	−0.0180 (11)	−0.0116 (12)
O25	0.0243 (13)	0.0319 (15)	0.0227 (13)	0.0050 (10)	−0.0088 (10)	−0.0165 (11)
C21	0.0207 (17)	0.031 (2)	0.018 (2)	0.0040 (15)	−0.0108 (15)	−0.0150 (17)
C22	0.0166 (16)	0.023 (2)	0.0174 (19)	0.0023 (14)	−0.0094 (14)	−0.0089 (16)
C23	0.0197 (17)	0.025 (2)	0.019 (2)	0.0029 (15)	−0.0101 (14)	−0.0063 (16)
C24	0.0224 (17)	0.026 (2)	0.020 (2)	0.0059 (15)	−0.0123 (15)	−0.0098 (17)
C25	0.0332 (19)	0.027 (2)	0.026 (2)	0.0100 (16)	−0.0141 (16)	−0.0182 (18)
C26	0.0324 (19)	0.023 (2)	0.019 (2)	0.0080 (16)	−0.0134 (16)	−0.0072 (16)
C27	0.0133 (16)	0.032 (2)	0.018 (2)	0.0027 (15)	−0.0095 (15)	−0.0108 (18)
C28	0.0168 (16)	0.019 (2)	0.0169 (19)	0.0011 (14)	−0.0049 (14)	−0.0056 (16)
C29	0.0225 (17)	0.027 (2)	0.0153 (19)	0.0048 (15)	−0.0104 (14)	−0.0064 (16)
C30	0.0248 (18)	0.024 (2)	0.0145 (19)	0.0017 (15)	−0.0107 (15)	−0.0047 (16)
C31	0.0281 (19)	0.022 (2)	0.025 (2)	0.0031 (15)	−0.0127 (16)	−0.0069 (17)
C32	0.0214 (17)	0.022 (2)	0.024 (2)	0.0050 (14)	−0.0103 (15)	−0.0094 (16)
B41	0.026 (2)	0.035 (3)	0.017 (2)	0.0007 (19)	−0.0083 (18)	−0.006 (2)
N41	0.0249 (16)	0.041 (2)	0.036 (2)	0.0050 (14)	−0.0090 (15)	−0.0192 (18)
O41	0.0315 (13)	0.0310 (15)	0.0220 (13)	0.0021 (11)	−0.0159 (10)	−0.0066 (12)
O42	0.0271 (12)	0.0286 (15)	0.0176 (13)	0.0018 (11)	−0.0084 (10)	−0.0058 (11)
O43	0.0302 (12)	0.0322 (15)	0.0197 (13)	−0.0023 (11)	−0.0093 (10)	−0.0033 (12)
O44	0.0433 (15)	0.0343 (17)	0.0420 (17)	0.0069 (12)	−0.0229 (12)	−0.0176 (14)
O45	0.0239 (12)	0.0344 (15)	0.0157 (13)	0.0035 (10)	−0.0053 (10)	−0.0084 (12)
C41	0.0215 (18)	0.030 (2)	0.023 (2)	−0.0005 (16)	−0.0094 (15)	−0.0066 (18)
C42	0.0211 (18)	0.031 (2)	0.017 (2)	0.0017 (15)	−0.0104 (15)	−0.0086 (17)
C43	0.0253 (18)	0.030 (2)	0.023 (2)	−0.0005 (16)	−0.0106 (16)	−0.0043 (17)
C44	0.0223 (18)	0.030 (2)	0.023 (2)	0.0009 (16)	−0.0073 (15)	−0.0087 (18)
C45	0.0225 (18)	0.038 (2)	0.023 (2)	−0.0017 (17)	−0.0076 (15)	−0.0110 (18)
C46	0.0229 (18)	0.037 (2)	0.021 (2)	0.0050 (16)	−0.0109 (15)	−0.0067 (18)
C47	0.0161 (18)	0.031 (2)	0.029 (2)	−0.0005 (16)	−0.0077 (16)	−0.0057 (19)
C48	0.0181 (17)	0.035 (2)	0.020 (2)	0.0043 (15)	−0.0048 (15)	−0.0120 (18)
C49	0.0242 (19)	0.035 (2)	0.033 (2)	0.0082 (17)	−0.0136 (16)	−0.0127 (19)
C50	0.033 (2)	0.031 (2)	0.045 (3)	0.0061 (17)	−0.0132 (19)	−0.015 (2)
C51	0.037 (2)	0.035 (3)	0.033 (2)	0.0121 (18)	−0.0164 (18)	−0.0112 (19)
C52	0.0264 (19)	0.038 (2)	0.027 (2)	0.0083 (17)	−0.0078 (16)	−0.0135 (19)
O61	0.0400 (14)	0.0447 (19)	0.0402 (19)	0.0037 (15)	−0.0133 (13)	−0.0038 (14)
O62	0.0376 (15)	0.0347 (18)	0.0381 (15)	0.0054 (12)	−0.0178 (12)	−0.0220 (13)

### Geometric parameters (Å, º)

**Table:**

B1—O1	1.469 (5)	C23—C24	1.379 (5)
B1—O2	1.533 (3)	C24—C25	1.391 (3)
B1—O5	1.456 (3)	C25—H25	0.950
B1—C8	1.604 (5)	C25—C26	1.381 (5)
N1—H1'	0.84 (3)	C26—H26	0.950
N1—C9	1.340 (4)	C28—C29	1.384 (5)
N1—C10	1.334 (3)	C28—C32	1.392 (3)
O1—C1	1.357 (4)	C29—H29	0.950
O2—C7	1.316 (4)	C30—H30	0.950
O3—C7	1.241 (3)	C30—C31	1.360 (5)
O4—H4'	0.840 (12)	C31—H31	0.950
O4—C4	1.377 (4)	C31—C32	1.406 (5)
O5—H5'	0.839 (6)	C32—H32	0.950
C1—C2	1.395 (3)	B41—O41	1.476 (5)
C1—C6	1.398 (5)	B41—O42	1.512 (3)
C2—C3	1.404 (4)	B41—O45	1.471 (3)
C2—C7	1.474 (5)	B41—C48	1.590 (5)
C3—H3	0.950	N41—H41'	0.84 (3)
C3—C4	1.381 (5)	N41—C49	1.342 (5)
C4—C5	1.392 (3)	N41—C50	1.347 (4)
C5—H5	0.950	O41—C41	1.359 (4)
C5—C6	1.375 (4)	O42—C47	1.315 (4)
C6—H6	0.950	O43—C47	1.247 (3)
C8—C9	1.390 (5)	O44—H44'	0.84 (3)
C8—C12	1.391 (3)	O44—C44	1.380 (4)
C9—H9	0.950	O45—H45'	0.839 (11)
C10—H10	0.950	C41—C42	1.376 (4)
C10—C11	1.374 (5)	C41—C46	1.397 (5)
C11—H11	0.950	C42—C43	1.395 (5)
C11—C12	1.390 (4)	C42—C47	1.480 (6)
C12—H12	0.950	C43—H43	0.950
B21—O21	1.467 (4)	C43—C44	1.389 (5)
B21—O22	1.539 (3)	C44—C45	1.384 (4)
B21—O25	1.446 (4)	C45—H45	0.950
B21—C28	1.609 (5)	C45—C46	1.380 (5)
N21—H21'	0.84 (2)	C46—H46	0.950
N21—C29	1.346 (5)	C48—C49	1.393 (6)
N21—C30	1.340 (3)	C48—C52	1.392 (4)
O21—C21	1.355 (4)	C49—H49	0.950
O22—C27	1.302 (4)	C50—H50	0.950
O23—C27	1.242 (3)	C50—C51	1.353 (6)
O24—H24'	0.840 (18)	C51—H51	0.950
O24—C24	1.378 (4)	C51—C52	1.394 (5)
O25—H25'	0.84 (3)	C52—H52	0.950
C21—C22	1.386 (3)	O61—H61A	0.840 (18)
C21—C26	1.399 (5)	O61—H61B	0.840 (14)
C22—C23	1.400 (4)	O62—H62A	0.84 (3)
C22—C27	1.475 (5)	O62—H62B	0.84 (3)
C23—H23	0.950		
			
O1—B1—O2	110.5 (3)	C24—C25—C26	121.3 (3)
O1—B1—O5	112 (3)	H25—C25—C26	119.0
O1—B1—C8	108.9 (3)	C21—C26—C25	119.1 (3)
O2—B1—O5	107.9 (3)	C21—C26—H26	120.0
O2—B1—C8	107.8 (3)	C25—C26—H26	120.0
O5—B1—C8	109.8 (3)	O22—C27—O23	120.0 (4)
H1'—N1—C9	117 (1)	O22—C27—C22	117.6 (3)
H1'—N1—C10	120 (1)	O23—C27—C22	122.4 (3)
C9—N1—C10	122.3 (3)	B21—C28—C29	123.4 (3)
B1—O1—C1	118.5 (2)	B21—C28—C32	120.9 (3)
B1—O2—C7	122.4 (2)	C29—C28—C32	115.7 (3)
H4'—O4—C4	106 (2)	N21—C29—C28	121.7 (3)
B1—O5—H5'	113.5 (6)	N21—C29—H29	119.0
O1—C1—C2	121.3 (3)	C28—C29—H29	119.0
O1—C1—C6	119.0 (3)	N21—C30—H30	120.0
C2—C1—C6	119.7 (3)	N21—C30—C31	119.8 (3)
C1—C2—C3	120.2 (3)	H30—C30—C31	120.0
C1—C2—C7	120.1 (3)	C30—C31—H31	121.0
C3—C2—C7	119.7 (3)	C30—C31—C32	118.5 (3)
C2—C3—H3	120.0	H31—C31—C32	121.0
C2—C3—C4	119.4 (3)	C28—C32—C31	121.9 (3)
H3—C3—C4	120.0	C28—C32—H32	119.0
O4—C4—C3	122.7 (3)	C31—C32—H32	119.0
O4—C4—C5	117.3 (3)	O41—B41—O42	110.4 (3)
C3—C4—C5	120.0 (3)	O41—B41—O45	111.9 (3)
C4—C5—H5	119.0	O41—B41—C48	108.8 (3)
C4—C5—C6	121.2 (3)	O42—B41—O45	103.7 (3)
H5—C5—C6	119.0	O42—B41—C48	108.9 (3)
C1—C6—C5	119.6 (3)	O45—B41—C48	113.1 (3)
C1—C6—H6	120.0	H41'—N41—C49	117 (2)
C5—C6—H6	120.0	H41'—N41—C50	120 (2)
O2—C7—O3	119.5 (3)	C49—N41—C50	122.2 (4)
O2—C7—C2	117.5 (3)	B41—O41—C41	114.8 (3)
O3—C7—C2	123.0 (3)	B41—O42—C47	118.5 (3)
B1—C8—C9	121.0 (3)	H44'—O44—C44	104 (2)
B1—C8—C12	123.6 (3)	B41—O45—H45'	119 (1)
C9—C8—C12	115.3 (3)	O41—C41—C42	121.5 (3)
N1—C9—C8	121.9 (3)	O41—C41—C46	119.8 (3)
N1—C9—H9	119.0	C42—C41—C46	118.6 (3)
C8—C9—H9	119.0	C41—C42—C43	121.3 (3)
N1—C10—H10	120.0	C41—C42—C47	119.2 (3)
N1—C10—C11	119.7 (3)	C43—C42—C47	119.2 (3)
H10—C10—C11	120.0	C42—C43—H43	120.0
C10—C11—H11	120.0	C42—C43—C44	119.3 (3)
C10—C11—C12	118.4 (3)	H43—C43—C44	120.0
H11—C11—C12	120.0	O44—C44—C43	122 (3)
C8—C12—C11	122.4 (3)	O44—C44—C45	118.3 (3)
C8—C12—H12	119.0	C43—C44—C45	119.7 (3)
C11—C12—H12	119.0	C44—C45—H45	120.0
O21—B21—O22	110.4 (3)	C44—C45—C46	120.5 (3)
O21—B21—O25	107.4 (3)	H45—C45—C46	120.0
O21—B21—C28	109.5 (3)	C41—C46—C45	120.5 (3)
O22—B21—O25	109.1 (3)	C41—C46—H46	120.0
O22—B21—C28	107.3 (2)	C45—C46—H46	120.0
O25—B21—C28	113.1 (3)	O42—C47—O43	119.8 (3)
H21'—N21—C29	116 (1)	O42—C47—C42	117.8 (3)
H21'—N21—C30	121 (1)	O43—C47—C42	122.4 (3)
C29—N21—C30	122.4 (3)	B41—C48—C49	120.2 (3)
B21—O21—C21	115.9 (3)	B41—C48—C52	124.5 (3)
B21—O22—C27	120.9 (2)	C49—C48—C52	115.2 (3)
H24'—O24—C24	105 (2)	N41—C49—C48	121.8 (4)
B21—O25—H25'	114 (2)	N41—C49—H49	119.0
O21—C21—C22	121.4 (3)	C48—C49—H49	119.0
O21—C21—C26	118.8 (3)	N41—C50—H50	120.0
C22—C21—C26	119.8 (3)	N41—C50—C51	119.5 (3)
C21—C22—C23	120.3 (3)	H50—C50—C51	120.0
C21—C22—C27	119.8 (3)	C50—C51—H51	120.0
C23—C22—C27	119.9 (3)	C50—C51—C52	119.1 (4)
C22—C23—H23	120.0	H51—C51—C52	120.0
C22—C23—C24	119.8 (3)	C48—C52—C51	122.2 (3)
H23—C23—C24	120.0	C48—C52—H52	119.0
O24—C24—C23	122.4 (3)	C51—C52—H52	119.0
O24—C24—C25	118.1 (3)	H61A—O61—H61B	108 (3)
C23—C24—C25	119.5 (3)	H62A—O62—H62B	116 (3)
C24—C25—H25	119.0		
			
O5—B1—O1—C1	−84.3 (3)	B21—O22—C27—O23	−172.6 (2)
O2—B1—O1—C1	36.0 (3)	B21—O22—C27—C22	8.1 (3)
C8—B1—O1—C1	154.2 (2)	C21—C22—C27—O23	−167.6 (2)
O5—B1—O2—C7	92.1 (3)	C23—C22—C27—O23	11.3 (4)
O1—B1—O2—C7	−30.6 (3)	C21—C22—C27—O22	11.6 (4)
C8—B1—O2—C7	−149.4 (2)	C23—C22—C27—O22	−169.5 (2)
B1—O1—C1—C2	−23.2 (4)	O25—B21—C28—C29	94.2 (3)
B1—O1—C1—C6	159.4 (2)	O21—B21—C28—C29	−146.0 (3)
O1—C1—C2—C3	−178.6 (2)	O22—B21—C28—C29	−26.2 (4)
C6—C1—C2—C3	−1.3 (4)	O25—B21—C28—C32	−83.3 (3)
O1—C1—C2—C7	0.4 (4)	O21—B21—C28—C32	36.4 (4)
C6—C1—C2—C7	177.8 (2)	O22—B21—C28—C32	156.3 (2)
C1—C2—C3—C4	1.0 (4)	C30—N21—C29—C28	0.5 (4)
C7—C2—C3—C4	−178.0 (2)	C32—C28—C29—N21	1.9 (4)
C2—C3—C4—O4	−178.0 (2)	B21—C28—C29—N21	−175.8 (3)
C2—C3—C4—C5	−0.3 (4)	C29—N21—C30—C31	−1.8 (4)
O4—C4—C5—C6	177.7 (2)	N21—C30—C31—C32	0.8 (4)
C3—C4—C5—C6	−0.1 (4)	C29—C28—C32—C31	−2.9 (4)
C4—C5—C6—C1	−0.1 (4)	B21—C28—C32—C31	174.8 (2)
O1—C1—C6—C5	178.2 (2)	C30—C31—C32—C28	1.6 (4)
C2—C1—C6—C5	0.8 (4)	O45—B41—O41—C41	69.1 (3)
B1—O2—C7—O3	−169.4 (2)	O42—B41—O41—C41	−45.8 (3)
B1—O2—C7—C2	10.5 (4)	C48—B41—O41—C41	−165.2 (2)
C1—C2—C7—O3	−174.1 (2)	O45—B41—O42—C47	−77.1 (3)
C3—C2—C7—O3	4.9 (4)	O41—B41—O42—C47	42.8 (4)
C1—C2—C7—O2	6.0 (4)	C48—B41—O42—C47	162.2 (2)
C3—C2—C7—O2	−175.0 (2)	B41—O41—C41—C42	21.8 (4)
O5—B1—C8—C9	74.1 (3)	B41—O41—C41—C46	−159.4 (3)
O1—B1—C8—C9	−163.0 (2)	O41—C41—C42—C43	−176.7 (3)
O2—B1—C8—C9	−43.1 (3)	C46—C41—C42—C43	4.6 (4)
O5—B1—C8—C12	−102.5 (3)	O41—C41—C42—C47	9.1 (4)
O1—B1—C8—C12	20.4 (4)	C46—C41—C42—C47	−169.7 (3)
O2—B1—C8—C12	140.3 (3)	C41—C42—C43—C44	−1.9 (4)
C10—N1—C9—C8	1.8 (4)	C47—C42—C43—C44	172.4 (3)
C12—C8—C9—N1	−0.8 (4)	C42—C43—C44—O44	179.3 (3)
B1—C8—C9—N1	−177.6 (3)	C42—C43—C44—C45	−1.9 (4)
C9—N1—C10—C11	−1.2 (4)	O44—C44—C45—C46	−178.2 (2)
N1—C10—C11—C12	−0.5 (4)	C43—C44—C45—C46	3.0 (4)
C10—C11—C12—C8	1.6 (4)	C44—C45—C46—C41	−0.2 (4)
C9—C8—C12—C11	−0.9 (4)	O41—C41—C46—C45	177.7 (2)
B1—C8—C12—C11	175.9 (3)	C42—C41—C46—C45	−3.5 (4)
O25—B21—O21—C21	−76.3 (3)	B41—O42—C47—O43	169.5 (2)
O22—B21—O21—C21	42.6 (3)	B41—O42—C47—C42	−14.0 (4)
C28—B21—O21—C21	160.5 (2)	C41—C42—C47—O43	163.3 (3)
O25—B21—O22—C27	83.4 (3)	C43—C42—C47—O43	−11.1 (4)
O21—B21—O22—C27	−34.4 (3)	C41—C42—C47—O42	−13.1 (4)
C28—B21—O22—C27	−153.7 (2)	C43—C42—C47—O42	172.5 (2)
B21—O21—C21—C22	−26.6 (4)	O45—B41—C48—C52	98.1 (3)
B21—O21—C21—C26	154.9 (3)	O41—B41—C48—C52	−26.9 (4)
O21—C21—C22—C23	179.0 (2)	O42—B41—C48—C52	−147.2 (3)
C26—C21—C22—C23	−2.5 (4)	O45—B41—C48—C49	−79.6 (4)
O21—C21—C22—C27	−2.1 (4)	O41—B41—C48—C49	155.4 (3)
C26—C21—C22—C27	176.4 (2)	O42—B41—C48—C49	35.0 (4)
C21—C22—C23—C24	−0.9 (4)	C50—N41—C49—C48	−2.0 (5)
C27—C22—C23—C24	−179.8 (3)	C52—C48—C49—N41	−0.8 (4)
C22—C23—C24—O24	−177.1 (2)	B41—C48—C49—N41	177.2 (3)
C22—C23—C24—C25	3.8 (4)	C49—N41—C50—C51	3.3 (5)
O24—C24—C25—C26	177.5 (2)	N41—C50—C51—C52	−1.8 (5)
C23—C24—C25—C26	−3.3 (4)	C50—C51—C52—C48	−1.0 (5)
C24—C25—C26—C21	−0.1 (4)	C49—C48—C52—C51	2.2 (4)
O21—C21—C26—C25	−178.4 (2)	B41—C48—C52—C51	−175.6 (3)
C22—C21—C26—C25	3.0 (4)		

### Hydrogen-bond geometry (Å, º)

**Table:**

*D*—H···*A*	*D*—H	H···*A*	*D*···*A*	*D*—H···*A*
O62—H62*A*···O25	0.84	1.86	2.702 (4)	174
O45—H45′···O23^i^	0.84	1.94	2.777 (2)	177
O4—H4′···O45^ii^	0.84	1.77	2.579 (3)	162
O61—H61*A*···O43^ii^	0.84	1.95	2.749 (3)	159
O5—H5′···O3^iii^	0.84	1.93	2.773 (2)	178
O24—H24′···O5^iv^	0.84	1.8	2.638 (3)	179
O25—H25′···O4^v^	0.84	1.95	2.791 (3)	173
O62—H62*B*···O24^vi^	0.84	2.02	2.810 (4)	157
O44—H44′···O61^vii^	0.84	1.82	2.656 (3)	177
O61—H61*B*···O62^viii^	0.84	1.83	2.673 (3)	178
N1—H1′···O23^i^	0.84	1.89	2.725 (4)	176
N21—H21′···O3^ix^	0.84	1.89	2.727 (4)	170
N41—H41′···O43^x^	0.84	1.92	2.749 (5)	169
C11—H11···O62^iv^	0.95	2.57	3.348 (4)	140
C23—H23···O5^iv^	0.95	2.59	3.215 (4)	123
C43—H43···O61^vii^	0.95	2.57	3.201 (4)	124

Symmetry codes: (i) *x*+1, *y*, *z*; (ii) *x*, *y*, *z*−1; (iii) −*x*+2, −*y*+1, −*z*; (iv) −*x*+1, −*y*+1, −*z*+1; (v) −*x*+1, −*y*+1, −*z*; (vi) −*x*, −*y*+2, −*z*+1; (vii) −*x*+1, −*y*, −*z*+1; (viii) *x*, *y*−1, *z*; (ix) *x*−1, *y*, *z*; (x) −*x*+1, −*y*+1, −*z*+2.
